# Contained abdominal aortic rupture secondary to follicular lymphoma invasion

**DOI:** 10.1016/j.jvscit.2022.03.015

**Published:** 2022-04-11

**Authors:** Melissa K. Meghpara, Albertina Sebastian, Mahmoud Almadani, Jennifer K. Henaghan, Yiwu J. Huang, Robert Y. Rhee

**Affiliations:** a Department of Vascular Surgery, Maimonides Medical Center, Brooklyn, NY; b Department of Anatomic Pathology, Maimonides Medical Center, Brooklyn, NY; c Department of Hematology/Oncology, Maimonides Medical Center, Brooklyn, NY

**Keywords:** Abdominal aorta, Abdominal aortic rupture, Follicular lymphoma

## Abstract

An 87-year-old woman with grade IIIb follicular lymphoma treated with rituximab had presented with nausea, emesis, and chest pain of 1 day duration. She was found to have a contained abdominal aortic rupture secondary to follicular lymphoma invasion. She safely and successfully underwent emergent endovascular aortic repair. We have described a rare case of extranodal disease in follicular lymphoma associated with abdominal aortic pseudoaneurysms, likely due to a combination of malignancy-induced chronic inflammation and radiation therapy and chemotherapy side effects.

Follicular lymphoma (FL) is the second most common subtype of non-Hodgkin lymphoma, accounting for 20% of all cases of non-Hodgkin lymphoma.^1^ Extranodal disease is rare in patients with FL, and the most common sites for disseminated disease have included the spleen, liver, and bone marrow.^2^ Few cases of rupture, aneurysm, and dissection of the thoracic aorta related to lymphoma have been reported.^3^^,^^4^ However, to the best of our knowledge, no studies have reported FL affecting the infrarenal abdominal aorta. We have described the case of a patient who had undergone endovascular repair of a contained infrarenal abdominal aortic rupture secondary to extranodal FL invasion.

## Case report

An 87-year-old woman had presented with nausea and emesis, followed by retrosternal chest pain of 1 day duration. Her medical history was significant for coronary artery disease, hypertension, atrial fibrillation, congestive heart failure, and grade IIIb follicular lymphoma. She had been previously treated with rituximab induction weekly for 4 weeks, followed by maintenance therapy for 2 years, and had completed treatment in January 2018. Her lymphoma had slowly progressed since 2019, with significant local progression in the same retroperitoneal region as the site of origin. The patient provided written informed consent for the report of her case details and imaging studies.

**Fig 1:**
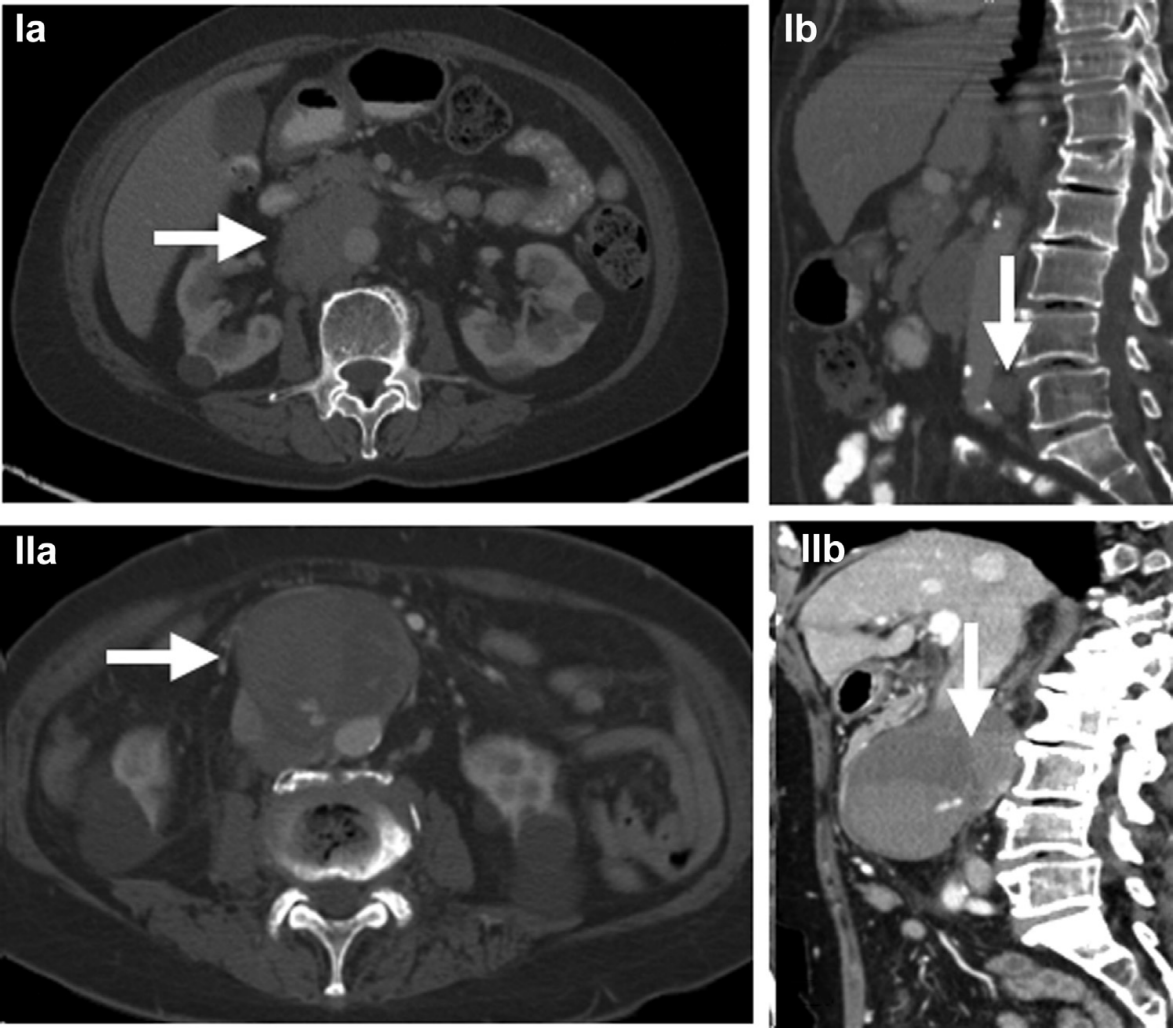
Computed tomography angiograms before rupture from 2019 with evidence of mild lymphadenopathy, in transverse (**Ia**; *arrow*) and coronal (**Ib**; *arrow*) views. Computed tomography angiograms after rupture showing evidence of significant lymphadenopathy with infrarenal aortic pseudoaneurysm and active bleeding, in both transverse (**IIa**; *arrow*) and coronal **(IIb)** views. Lymphoma (*arrow*) can be differentiated from rupture in **IIa**.

On presentation to the emergency room, on April 5, 2021, she was hypertensive with significant pain. Computed tomography angiography demonstrated a 6.5 × 5.7-cm infrarenal pseudoaneurysm in the region of previously seen adenopathy that was concerning for a contained aortic rupture secondary to the lymphoma (Fig 1). The patient was emergently taken to the operating room for endovascular repair of the aorta.

**Fig 2:**
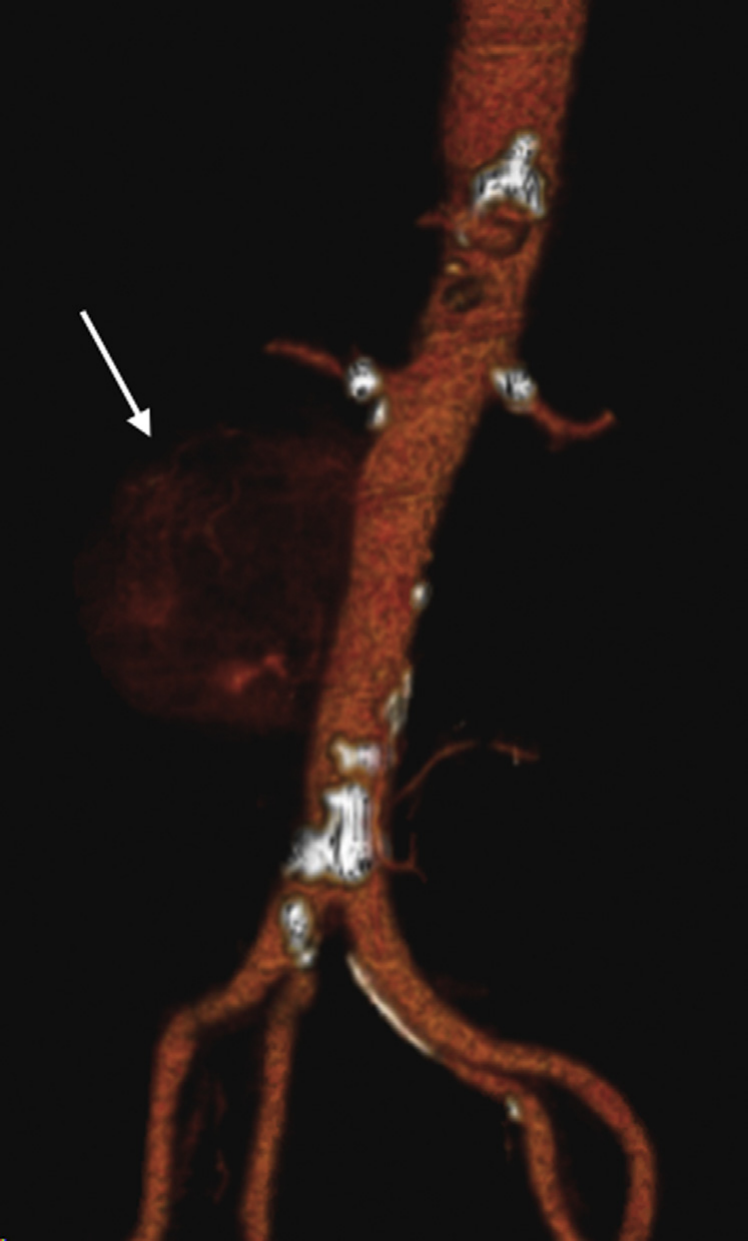
Three-dimensional reconstruction of computed tomography angiogram demonstrating infrarenal aortic pseudoaneurysm with a focus of active bleeding (*arrow*).

**Fig 3:**
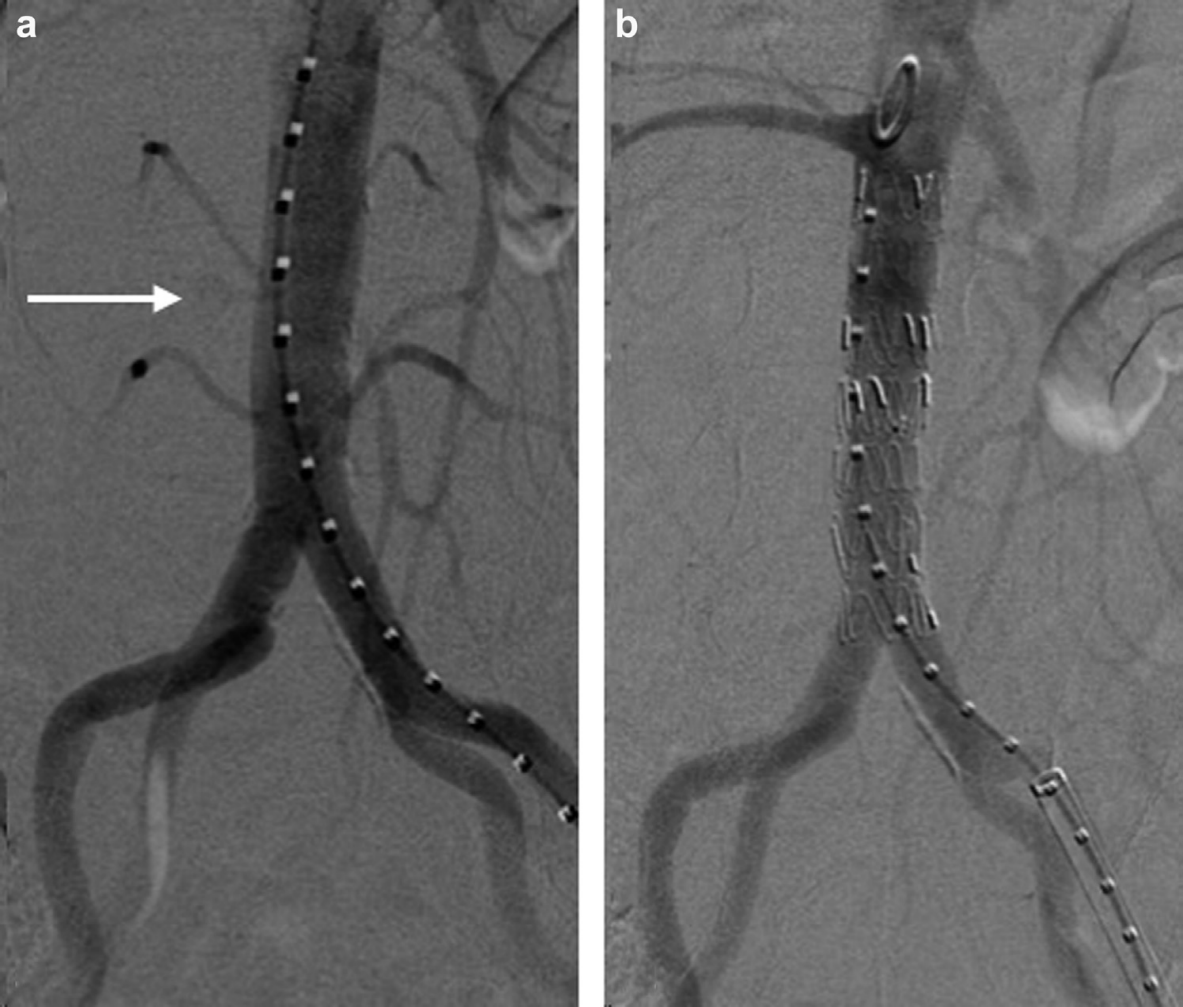
Intraoperative fluoroscopic views demonstrating the point of active bleeding (*arrow*) before graft deployment **(a)** and resolution of bleeding after covered stent placement **(b)**.

Percutaneous access was established via the left common femoral artery, and aortography confirmed an aortic rupture at the midportion of the infrarenal aorta (Fig 2). A 20-mm × 4.5-cm Excluder conformable abdominal aortic aneurysm endoprosthesis aortic extender (W.L. Gore & Associates, Flagstaff, AZ) was deployed at the rupture site just above the aortic bifurcation. To obtain an adequate seal and protect the infrarenal aorta, an additional 20-mm × 4.5-cm cuff was deployed proximally, followed by a 23-mm × 4.5-cm cuff up to the level of the renal arteries. Completion angiography confirmed adequate placement of the endoprosthesis without evidence of an endoleak (Fig 3). Aortic duplex ultrasound on postoperative day 1 demonstrated no evidence of endoleak with an avascular mass present between the inferior vena cava and aorta, likely the lymphoma. The patient’s postoperative course was uneventful, and she was discharged on postoperative day 3. At 1 month of follow-up, the patient was doing well, and a repeat duplex ultrasound scan had demonstrated resolving retroperitoneal hematoma. In addition, the patient had undergone post-stenting radiation therapy to the periaortic lymphoma and tolerated it well without any issues.

## Discussion

Abdominal aortic pseudoaneurysms occur infrequently in clinical practice.^5^ Although the typical diagnosis of acute ruptured aneurysms and pseudoaneurysms of the aorta involve a triad of chest and/or back pain, a pulsatile abdominal mass, and hypotension, it is not uncommon for a patient to lack all the features of this triad.^6^ The more commonly associated etiologies include trauma, infection, and inflammation. Rarely, pseudoaneurysms can be caused by chronic inflammation involved with malignancy.

Lymphomas are the most common type of blood neoplasm and can lead to secondary involvement of the aorta. By inflammatory cytokine release, lymphomatous tissue can lead to aneurysmal degeneration and weakening of the vessel wall.^7^^,^^8^ Disruption of the aortic wall causes dissection of blood within the vessel layers and, ultimately, leads to formation of a perfused sac communicating directly with the aortic lumen.^9^

Prior treatment with chemotherapeutic agents is also thought to affect vessel wall strength. In a 2017 study by Leopardi et al,^10^ patients with aortic aneurysms diagnosed during computed tomography for oncologic follow-up were analyzed retrospectively according to the oncologic treatment they had received. Those undergoing chemotherapy were found to have significant aneurysmal growth and a higher rate of rupture compared with those not undergoing oncologic treatment.^10^ Chemotherapy regimens that specifically include rituximab have also been reported to contribute to abdominal aortic wall stress.^11^ In the present case, the patient had a known malignant adenopathy present within the periaortic window and the aortocaval window without a history of an aortic aneurysm. The combination of malignant lymphocytic infiltration in an already weakened vessel wall secondary to oncologic treatment might have been responsible for the contained aortic rupture in our patient.

In contrast to true abdominal aortic aneurysms, pseudoaneurysms are classified as false because they generally lack all layers of the vessel wall. The form they subsequently develop is secondary to the products of the clotting cascade, which form a wall and lead to containment of blood.^12^ The lack of the robustness of a real vessel wall means that these pseudoaneurysms are fragile and must be treated without delay, regardless of their size.^13^

A variety of options exist for the treatment of pseudoaneurysms. Open surgical repair consists of pseudoaneurysm resection with primary repair, graft interposition, or patch aortoplasty. When open repair is not feasible, because of comorbidities or hostile abdominal anatomy, an endovascular approach can be pursued, such as exclusion using covered stents.^14^

For our patient, we chose to treat the pseudoaneurysm using Excluder conformable abdominal aortic aneurysm endoprosthesis aortic extender cuffs (W.L. Gore & Associates). Deployment of three separate devices was performed to adequately cover the entire area affected by the lymphoma, which was the total length of the infrarenal aorta. The advantage of using aortic extension cuffs, instead of a thoracic endograft, was the shorter length of the graft. This allowed us to precisely cover the weakened surface area of the vessel. In addition, the novel Food and Drug Administration–approved conformable device has the ability to angulate, or “conform,” the proximal end of the graft before deployment. This allows for use of the entire surface area of the available proximal sealing zone. Furthermore, its independent sealing zones eliminate the need for extensive graft overlapping, without concern for an increased risk of endoleaks.^15^

Although direct tumor erosion or pressure necrosis secondary to a mass effect can lead to aortic rupture, it is important to recognize that the variations in hemodynamic parameters caused by systemic chemotherapy can also contribute to weakening of the aortic wall. Over time, this weakening can lead to formation of aneurysms and pseudoaneurysms and, eventually, rupture of the aorta. These complications have been rare in the setting of lymphoma; however, in our experience, they can be safely and effectively treated using an endovascular approach. It is important to consider abdominal aortic pseudoaneurysms as a potential entity in the differential diagnosis for patients with a history of lymphoma who present with chest or back pain.
